# Precocity a Sign of Inferiority

**Published:** 1881-05

**Authors:** 


### PRECOCITY A SIGN OF INFERIORITY.

M. D. Delaunay, in a communication to the French Societe de Biologie, has advanced the opinion that precocity is a sign of biological inferiority. In support of his position, he adduces the fact that the lower species develope more rapidly, and are at the same time more precocious than those higher in the scale. Man is the longest of all in arriving at maturity; and the inferior races of men are more precocious than the superior, as is seen in the children of the Esquimaux, Negroes, Cochin-Chinese, Japanese, Arabs, etc., who are, up to a certain age, more vigorous and more intellectual than small Europeans. Precociousness becomes less and less in proportion to the advance made by any race in civilization—a fact which is illustrated by the lowering of the standard for recruits, which has been made necessary in France twice during the present century, by the decreasing rapidly of growth of the youth of the country. Women are more precocious than men, and in all domestic animals the female is formed sooner than the male. From eight to twelve years of age a girl gains one pound a year on a boy, and in mixed schools girls obtain the first places up to the age of twelve. The inferior tissues and organs develop before the higher ones, and the brain is the slowest of all organs to develop. M. Delaunay concludes his paper by stating that the precocity of organs and organisms is in an inverse ratio to the extent of their evolution.

